# Sonochemical Synthesis of Magnetite/Poly(lactic acid) Nanocomposites

**DOI:** 10.3390/polym15244662

**Published:** 2023-12-11

**Authors:** Juliene Oliveira Campos de França, Quezia dos Santos Lima, Mariana Martins de Melo Barbosa, Ana Lívia Fernandes Fonseca, Guilherme de França Machado, Sílvia Cláudia Loureiro Dias, José Alves Dias

**Affiliations:** Laboratory of Catalysis, Chemistry Institute (IQ-UnB), University of Brasília, Campus Universitário Darcy Ribeiro–Asa Norte, Brasília 70910-900, DF, Brazil; julienechemistry@gmail.com (J.O.C.d.F.); quezia.sl198@gmail.com (Q.d.S.L.); marimartins.melo@hotmail.com (M.M.d.M.B.); analiviaffonseca@gmail.com (A.L.F.F.); guilhermedefrancamachado@gmail.com (G.d.F.M.); scdias@unb.br (S.C.L.D.)

**Keywords:** magnetite, magnetic nanoparticles (MNP), poly(lactic acid)-PLA, polymerization based on D,L-lactic acid, nanocomposites MNP-PLA, sonochemical synthesis

## Abstract

Nanocomposites based on poly(lactic acid) (PLA) and magnetite nanoparticles (MNP-Fe_3_O_4_) show promise for applications in biomedical treatments. One key challenge is to improve the stabilization and dispersion of MNP-Fe_3_O_4_. To address this, we synthesized MNP-Fe_3_O_4_/PLA nanocomposites using ultrasound mediation and a single iron(II) precursor, eliminating the need for surfactants or organic solvents, and conducted the process under ambient conditions. The resulting materials, containing 18 and 33 wt.% Fe_3_O_4_, exhibited unique thermal behavior characterized by two mass losses: one at a lower degradation temperature (T_d_) and another at a higher T_d_ compared to pure PLA. This suggests that the interaction between PLA and MNP-Fe_3_O_4_ occurs through hydrogen bonds, enhancing the thermal stability of a portion of the polymer. Fourier Transform Infrared (FT-IR) analysis supported this finding, revealing shifts in bands related to the terminal –OH groups of the polymer and the Fe–O bonds, thereby confirming the interaction between the groups. Raman spectroscopy demonstrated that the PLA serves as a protective layer against the oxidation of MNP-Fe_3_O_4_ in the 18% MNP-Fe_3_O_4_/PLA nanocomposite when exposed to a high-power laser (90 mW). Transmission Electron Microscopy (TEM) and Scanning Electron Microscopy (SEM) analyses confirmed that the synthetic procedure yields materials with dispersed nanoparticles within the PLA matrix without the need for additional reactants.

## 1. Introduction

Polymeric nanocomposites composed of poly(lactic acid) (PLA) and magnetite nanoparticles (MNP-Fe_3_O_4_) are very attractive materials from the perspective of biomedical applications because of their biocompatible attributes. Both components are considered non-toxic and offer features beneficial for drug delivery systems, hyperthermic treatments, and as contrast agents in magnetic resonance imaging (MRI). Beyond biomedical utilities, these composites may also possess adsorptive characteristics useful for air filtration and dye adsorption [1,2].

Strategies for improving the stabilization and dispersion of nanoparticles often involve the functionalization of magnetite with polymeric substances and surfactants, as extensively documented in the literature [3,4]. Such approaches also enhance the compatibility and miscibility of magnetite within the PLA polymer matrix [5]. A majority of the reviewed studies employ techniques involving a mixture of magnetite functionalized with oleic acid or similar substances, primarily using emulsion and melt-mixing methodologies to fabricate the composites.

Various studies have reported the synthesis and characterization of these composites through diverse methods. These include single or double emulsion, typically with magnetite functionalized by oleic acid [6,7,8,9,10,11,12,13,14]; microwave-induced synthesis involving oleic acid-coated magnetite [15]; ozone-mediated techniques featuring magnetite functionalized with maleimide benzoic acid (MBA) [16]; melt compounding synthesis using magnetite functionalized with polymethylhydrogensiloxane (MHX) [17]; as well as blow fusion [1], extrusion [18], doctor-blade [19], solvent-casting [20,21,22,23,24], matrix-assisted pulsed laser evaporation (MAPLE) [25], and other melt mixing methods [26]. A brief summary of these works is provided in Table 1.

It is important to mention that Pigareva et al. [27] prepared micelles of poly(D,L-lactide)-b-(ethylene glycol methyl ether) diblock copolymer (PLA–PEG) with a mean diameter of 20 nm and enhanced the dispersion of maghemite nanoparticles (MNPs) using ultrasound to obtain micelles with a strong magnetic response. Thus, magneto-responsive PLA–PEG micelles were obtained that were capable of encapsulating hydrophobic drugs (paclitaxel, PTX), which is a highly effective anticancer therapeutic agent. The incorporation of MNP did not change the size and morphology of the micelles. An interesting point here is that under the sonication treatment, the MNPs could improve the stabilization of the PLA hydrophobic surface.

Also, it is noteworthy that the synthesis of such materials, as described in the cited articles, commonly involves the use of organic solvents—generally chlorinated—or high-temperature processes. These factors likely contribute to increased operational costs and time. A more sustainable alternative offering energy efficiency and eliminating the need for organic solvents is sonochemical synthesis.

Sonochemical syntheses have proven to be effective in producing polymeric composites with inorganic fillers. This technique often leads to excellent dispersion of the inorganic component within the selected matrices, enhancing their mechanical and thermal properties. For example, Balachandramohan et al. [28] successfully produced Fe_3_O_4_/guargum nanocomposites with a diameter of 48 nm by introducing polysaccharide into an aqueous ferrous sulfate solution and using sodium hydroxide (NaOH) as a base. This was conducted under a continuous flow of N_2_ for one hour and subjected to ultrasonic irradiation. Ghanbari et al. [29] synthesized Fe_3_O_4_ nanoparticles (approximately 60 nm in diameter) and Fe_3_O_4_/PVA (polyvinyl alcohol) nanocomposites using a straightforward technique, employing FeCl_2_ as the precursor without the need for surfactants or inert gas, all at room temperature. Additional recent publications also highlight the synthesis of polymer nanocomposites exhibiting superior thermal and/or mechanical properties through sonochemical methods, using either one or two steps [30,31,32,33,34,35,36,37].

Therefore, the aim of the present study is to provide a rapid and straightforward sonochemical synthesis of MNP-Fe_3_O_4_/PLA nanocomposites. This approach will utilize a single iron precursor (FeSO_4_) and operate without the need for surfactants or organic solvents under ambient conditions and at room temperature. Subsequently, the synthesized materials will undergo characterization in terms of their structure (XRD, FT-IR, Raman), morphology (TEM and SEM), composition (EDX), and thermal properties (TG/DTG).

## 2. Experimental Procedures

### 2.1. Polymer Synthesis

**Figure 1:**
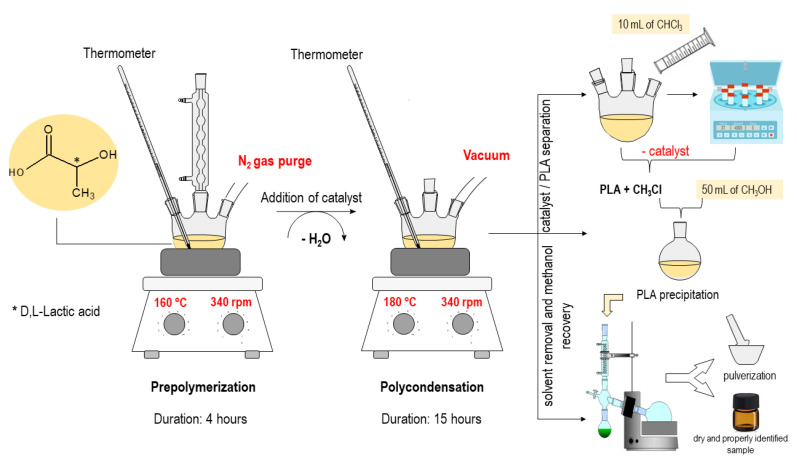
Schematic description of PLA synthesis.

Poly(lactic acid) (PLA) was synthesized through direct polycondensation of D,L-lactic acid, employing a commercial silica-alumina catalyst (Sigma-Aldrich, USA, SiO_2_/Al_2_O_3_ mole ratio = 12.4), which was activated to a protonic form after treatment at 550 °C for 8 h. The synthesis process comprises two steps: pre-polymerization and catalytic polycondensation. In the pre-polymerization stage, water is removed from a system containing D,L-lactic acid (85%, Vetec, Rio de Janeiro, Brazil) by heating the mixture to 160 °C, which is subjected to magnetic stirring at 340 rpm and undergoes distillation under N_2_ gas flow for 4 h. Then, the temperature is elevated to 180 °C, and the catalyst is introduced into the reaction medium. The distillation apparatus is then disassembled, and a vacuum hose is connected to the system, which is subsequently sealed hermetically and maintained in this state for an additional 15 h. Following the polycondensation, the catalyst is separated, and the polymer is crystallized through the addition of methanol. Ultimately, methanol and chloroform are eliminated through rotary evaporation. The polymer is then removed from the inner surfaces of the flask and pulverized using a mortar and pestle until it reaches a thoroughly dry PLA powder. For the purpose of comparison, a pre-polymer sample and PLA without the use of a catalyst were synthesized. Additionally, three PLA samples were obtained using the catalytic polycondensation method, employing silica-alumina as the catalyst, and they were named PLA 1X, PLA 2X, and PLA 3X. The detailed procedure has been described in references [38,39]. Figure 1 schematically outlines the aforementioned process.

### 2.2. Sonochemical Synthesis of MNP-Fe_3_O_4_/PLA Nanocomposites and MNP-Fe_3_O_4_

**Figure 2:**
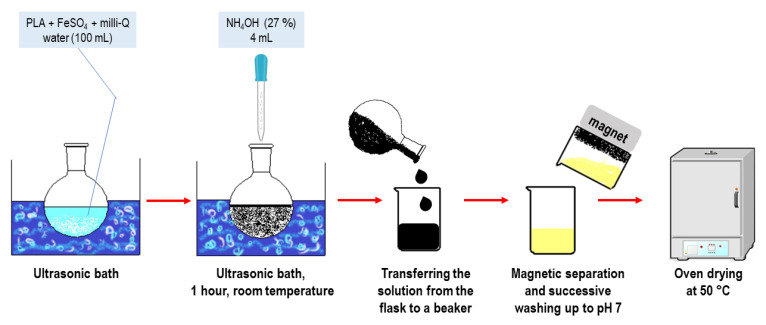
Sonochemical synthesis of MNP-Fe_3_O_4_/PLA nanocomposites and MNP-Fe_3_O_4_.

The sonochemical synthesis of MNP-Fe_3_O_4_/PLA nanocomposites was conducted by adding first PLA (synthesized in our laboratory) followed by ferrous sulfate (FeSO_4_·7H_2_O, Vetec, Rio de Janeiro, Brazil) to a round-bottom flask containing 100 mL of milli-Q water (Merck Millipore, model Direct 8, Guyancourt, France). The flask was placed in an ultrasonic bath (SolidSteel, model SSBuc-6L, São Paulo, Brazil, operating at 40 kHz) at room temperature (25 °C). The loadings were 20 and 35 wt.% of Fe_3_O_4_. For the preparation of 1 g of the composite, it was used 0.40 g of ferrous sulfate and 0.80 g of PLA; 0.70 g of ferrous sulfate and 0.65 g of PLA to obtain 20% and 35% MNP-Fe_3_O_4_/PLA nanocomposites, respectively. Subsequently, 4 mL of aqueous ammonia solution (27 wt.%, Merck, São Paulo, Brazil) was added dropwise into this mixture of PLA and ferrous sulfate. Upon the addition of ammonia, a black precipitate of magnetite was visibly formed. The reaction was allowed to proceed for 1 h under ultrasonic radiation. After that, the suspension was transferred to a beaker, and the nanocomposite was isolated via magnetic separation using a neodymium magnet. These nanoparticles were successively rinsed with milli-Q water until the pH reached 7 and subsequently dried in an oven (Ethik, model 400, São Paulo, Brazil) at 50 °C. Each synthesis was repeated three times, and the average yield was 60 to 65%, based on the expected mass of PLA plus magnetite. The process is schematically depicted in Figure 2.

Pure MNP-Fe_3_O_4_ was synthesized using a similar methodology but without PLA addition, according to the literature [40]. In a round-bottom flask immersed in the ultrasonic bath (40 kHz, 25 °C), 0.50 g of ferrous sulfate was dissolved in 100 mL of milli-Q water. After complete dissolution of the iron salt, 4 mL of 27 wt.% aqueous ammonia was added dropwise to the solution, resulting in a dark green solution that turned black after a few minutes of sonication. The solution was kept in the ultrasonic bath for 1 h, leading to the formation of a black magnetic precipitate (i.e., magnetite). The MNP was separated from the solution using a neodymium magnet. The nanoparticles were washed with milli-Q water several times until reaching a pH of 7. Finally, the nanoparticles were dried in an oven at 50 °C for 4 h. The synthesis was repeated three times (MNP-Fe_3_O_4_(1), MNP-Fe_3_O_4_(2), and MNP-Fe_3_O_4_(3)), and the products were characterized by powder X-ray diffraction (XRD) to confirm the formation of magnetite. The average yield was between 62 and 67%.

### 2.3. Characterization Methods

#### 2.3.1. Thermogravimetric Analysis (TG)

Thermogravimetric analyses were carried out on a TG analyzer (Shimadzu, model DTG-60H, Kyoto, Japan). Approximately 10 mg of sample was used in a platinum crucible subjected to a heating rate of 10 °C min^−1^ from room temperature (25 °C) up to 600 °C and a flow rate of 30 mL min^−1^ of N_2_ gas (White Martins, 99.999% purity, Rio de Janeiro, Brazil). The samples were pure PLA, MNP-Fe_3_O_4_, and MNP-Fe_3_O_4_/PLA composites. In the analysis for determination of the Fe_3_O_4_ residue, the same conditions were used, but synthetic air (White Martins, ±20% O_2_ and ±80% N_2_, 99.999% purity, Rio de Janeiro, Brazil) instead of gaseous N_2_.

#### 2.3.2. Fourier-Transform Infrared Spectroscopy (FT-IR)

FT-IR spectra were obtained using a spectrometer (Thermo Fisher Scientific, Nicolet, model 6700, Madison, WI, USA). The conditions were: 256 scans, a resolution of 4 cm^−1^, and transmittance mode using KBr pellets (1 wt.%).

#### 2.3.3. Raman Spectroscopy

Raman spectra were obtained using a spectrometer (Horiba, model LabRAM HR Evolution, Villeneuve-d’Ascq, France) under ambient conditions (25 °C). It was used with a laser at 795 nm and 90 mW of power, 64 acquisitions, and a resolution of 2 cm^−1^.

#### 2.3.4. Powder X-ray Diffraction (XRD)

Powder diffraction patterns were obtained in a diffractometer (Bruker, model D8 Focus, Karlsruhe, Germany). The conditions included CuKα tube (λ = 0.15418 nm), operating at 40 kV and 30 mA, and a scanning rate of 1° min^−1^ at 0.05° increments.

#### 2.3.5. Microscopy Analyses

The samples were analyzed by scanning electron microscopy (SEM) and transmission electron microscopy (TEM). The SEM images were obtained on a microscope (Jeol, model JSM-6610, Tokyo, Japan). TEM images were acquired with a transmission electron microscope (Jeol, model JEM 2100, Tokyo, Japan) operating at 200 kV. The powder was dispersed in ethyl alcohol using an ultrasonic bath, followed by placing it on a copper grid. The SEM microscope was equipped with energy dispersive X-ray spectroscopy (EDX) from Thermo Scientific NSS Spectral Imaging.

#### 2.3.6. Proton Nuclear Magnetic Resonance (^1^H NMR)

The proton nuclear magnetic resonance (^1^H NMR) spectra were obtained for PLA on CDCl_3_ using a spectrometer (Bruker, Avance III HD-Ascend model, at 14.1 T, 600 MHz for ^1^H, Ettlingen, Germany). The acquisition conditions included: i) ^1^H, 600 MHz, single pulse of 4.5 μs duration, acquisition time of 0.1 s, interval between pulses of 1 s, minimum of 20 acquisitions, and internal reference of TMS (δ = 0.0 ppm).

#### 2.3.7. Gel Permeation Chromatography 

Gel permeation chromatography (GPC) profiles were attained for PLA at 40 °C in equipment (Malvern, model Viscotek Rimax, Malvern, United Kingdon) equipped with a refractive index detector, a 60-position autosampler, and three GPC columns (8 mm × 30 cm) in KF-802.5, KF-804L, and KF-805L ovens. The mobile phase was eluted at 1 mL min^−1^ with THF (Aldrich, inhibitor-free for HPLC, 99.9%, Wyoming, USA). Before injection, the samples were prepared using 1.5–2.0 mg of PLA per mL of THF and filtered through a 0.45 μm membrane filter. Polystyrene was used to calibrate the system.

## 3. Results and Discussion 

### 3.1. Analyses of PLA Polymer and MNP-Fe_3_O_4_

Three PLA samples were synthesized to confirm the reproducibility of the polycondensation method for polymer production. The GPC results (Table 2) demonstrate that the synthesized materials exhibited quite similar molecular weights, even higher than those obtained for the polymer synthesized without a catalyst and for the pre-polymer, confirming our previous studies of this method [38,39]. This indicates that the silica-alumina catalyst favors the growth of PLA polymer chains. The molar masses of the polymers obtained in three runs are very close, making it possible to infer that the catalytic synthesis of PLA is reproducible in terms of polymer chain growth, especially when observing the values of M_n_ in Table 2.

**Figure 3:**
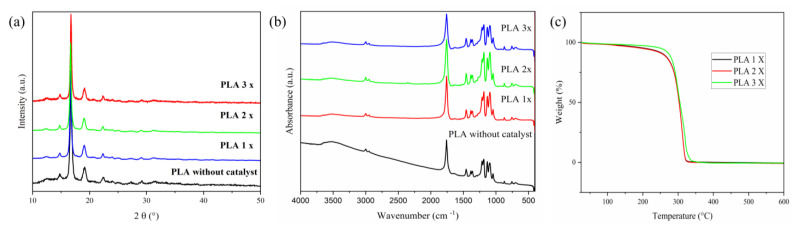
Characterization of PLA by: (**a**) XRD, (**b**) FT-IR, and (**c**) TG.

The X-ray diffraction (XRD) patterns of PLA samples (Figure 3a) are representative of either PLLA or PDLA crystalline structures. The semicrystalline pattern obtained for PLA was observed in the polymers synthesized with the silica-alumina catalyst, demonstrating an enantioselective character of the catalyst, as already discussed in the references [38,39]. The diffraction peaks observed at 2*θ* values of 14.7°, 16.6°, 19.1°, and 22.3° are indicative of PLLA homocrystals, whereas the peaks at 12.4° and 29.1° are characteristic of stereocomplex crystals (sc) [38,39,40,41,42,43]. These results are corroborated by polarimetry, which suggests an enantiomeric excess of 86% of the L-isomer in the PLA sample [44]. Specifically, the peaks at 2*θ* = 16.6° and 19.1° correspond to the hkl planes (110/200) and (203), respectively. Similarly, the peaks at 2*θ* = 14.8° and 22.3° can be ascribed to the hkl planes (010) and (015), respectively [39].

The FT-IR spectra of pure PLA samples (Figure 3b) exhibit an absorption band near 3506 cm^−1^, associated with the stretching of the –OH bond. Bands at 2997 and 2947 cm^−1^ correspond to the symmetric and asymmetric stretches of the –CH_3_ group. A very strong band at 1758 cm^−1^ is attributed to the stretching of the C=O bond. Additionally, the band at 1457 cm^−1^ represents the asymmetric bending of the –CH_3_ group, while the band at 1214 cm^−1^ is indicative of the asymmetric stretching of the C–O–C group. Another band at 1094 cm^−1^ is related to the symmetric stretching of the same C–O–C group. A band at 756 cm^−1^ is attributed to the bending of the C=O bond [45,46,47]. In the pure PLA sample, an absorption band at 3506 cm^−1^ is indicative of the characteristic terminal –OH bond found in low-molecular-weight polymers [48].

The TG curves of PLA (Figure 3c) show that all samples undergo maximum thermal degradation at temperatures (T_d_) around 315 °C and exhibit a single region of mass loss.

**Figure 4:**
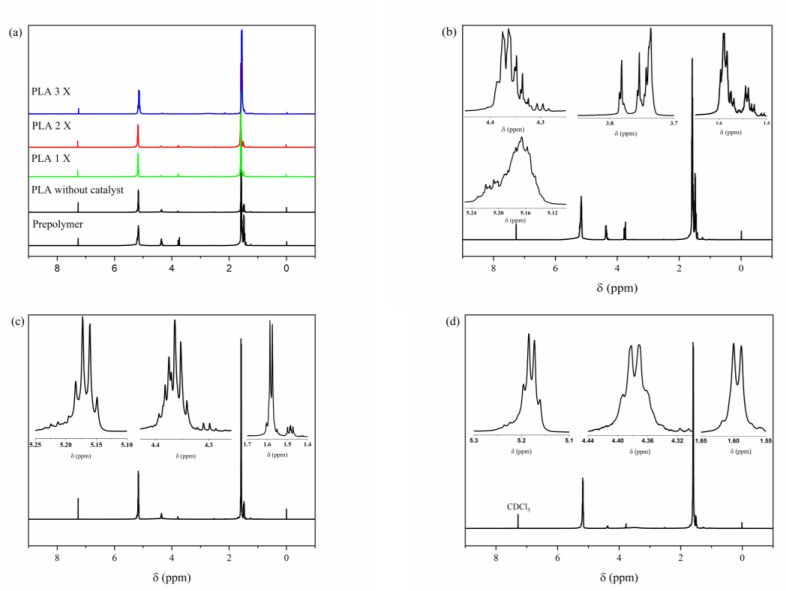
¹H NMR spectra of the samples: (**a**) pre-polymer; PLA without catalyst; PLA samples synthesized using silica-alumina catalyst. (**b**) ¹H NMR spectrum of the pre-polymer PLA. (**c**) PLA is synthesized without a catalyst. (**d**) PLA synthesized using a silica-alumina catalyst (sample 3X).

The ¹H NMR spectra of the pre-polymer, catalyst-free PLA, and PLA 3X samples are presented in Figure 4a. The pre-polymer (Figure 4b) exhibits multiplet signals between 1.4 and 1.6 ppm, between 3.7 and 3.8 ppm, 4.2 ppm, and also between 5 and 5.3 ppm, indicating a low degree of optical purity of the material. Additionally, there is a possibility of residual solvent and unreacted monomer presence, as pure polymer (e.g., PLA) typically displays a well-defined quartet in the region between 5 and 5.3 ppm, while racemic polymer shows overlapping signals, as depicted in references [49,50]. The ¹H NMR spectrum of catalyst-free PLA in Figure 4c showed more well-defined resonance signals, but it is still apparent that the signals appear overlapped, especially those related to the polymer, such as the signal between 5.1 and 5.2 ppm, associated with the methine CH-protons of the repeating units of PLA. The resonance signal between 4.2 and 4.4 ppm is related to a methine -CH group linked to a terminal hydroxyl group.

The ¹H NMR spectrum of the PLA 3X sample (Figure 4d) shows resonance signals characteristic of PLLA, including a well-defined quartet at approximately 5.2 ppm, corresponding to the CH– group of the polymer chain repeating units, and signals at 1.6 ppm and 4.3 ppm, corresponding, respectively, to the methyl –CH_3_ groups and the methine (CH) proton linked to a terminal –OH group [50,51,52]. These results indicate that, in addition to increasing the molar mass of the polymer, the silica-alumina catalyst also promotes greater optical purity of the synthesized materials. This observation is consistent with and supported by polarimetry results from a series of catalysts previously studied by the group [38,39,44].

**Figure 5:**
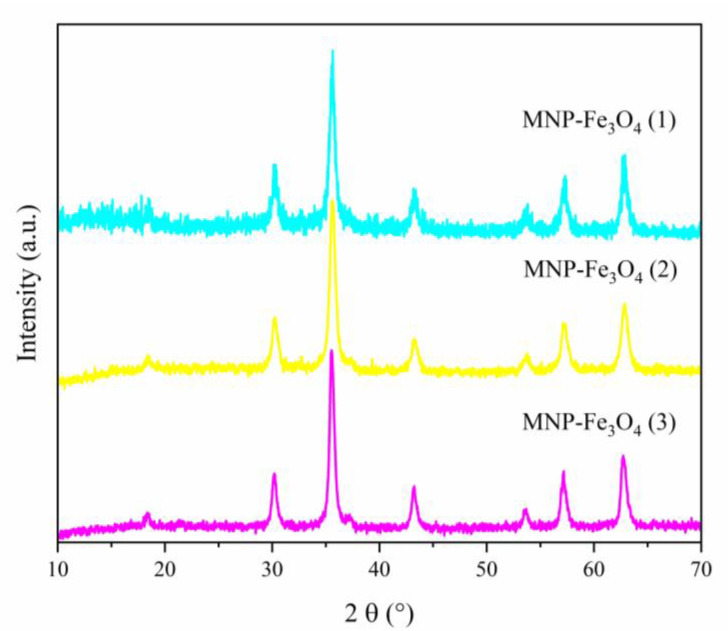
XRD of MNP-Fe_3_O_4_ samples synthesized via the sonochemical method.

The crystalline structure of MNP-Fe_3_O_4_ samples was examined by powder XRD (Figure 5). The obtained patterns displayed a high level of agreement with the crystallographic data of magnetite (PDF number 01-71-6337, ICDD). It can be numbered six characteristic peaks at 2θ = 30.27° (220); 35.71° (311); 43.44° (400); 53.91° (422); 57.36° (511); and 62.96° (440) for the MNP-Fe_3_O_4_. Clearly, the three patterns show the high reproducibility of the synthetic method.

### 3.2. Thermal Analysis

The thermogravimetric (TG) curve for MNP-Fe_3_O_4_ reveals two distinct mass loss events: one around 80 °C and the other at 235 °C, accounting for a total of 4% of the sample mass. The initial mass loss at approximately 80 °C is likely attributable to the desorption of physically adsorbed water from the surface of the magnetic nanoparticles. The subsequent mass loss at 235 °C is most probably related to chemically adsorbed hydroxyl groups or ammonium ions on the nanoparticle surface [53,54].

**Figure 6:**
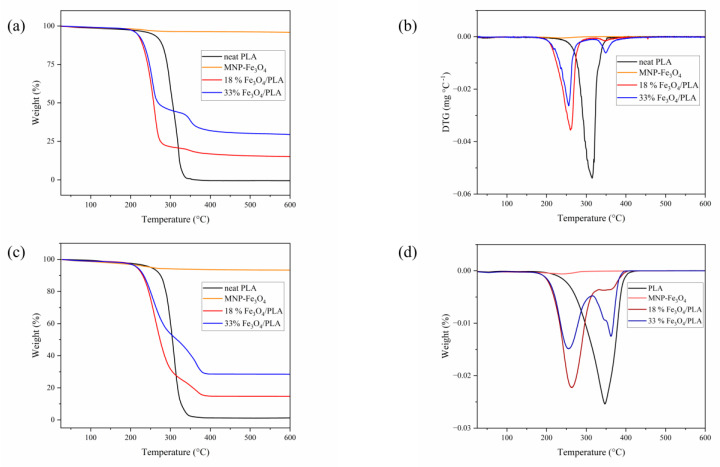
TG (**a**) and DTG (**b**) curves of pure PLA, MNP-Fe_3_O_4_, and composites of 18% and 33% MNP-Fe_3_O_4_/PLA, obtained under N_2_ atmosphere. TG (**c**) and DTG (**d**) curves of pure PLA, MNP-Fe_3_O_4_, and composites of 18% and 33% MNP-Fe_3_O_4_/PLA, obtained under synthetic air.

As illustrated in Figure 6, the thermal degradation of neat PLA started at roughly 230 °C, reaching its peak degradation rate (T_d_) at 313 °C. The total mass loss is 100%, indicating complete decomposition of the polymer up to 600 °C, which is the maximum temperature at which the thermogravimetric analysis was conducted. Based on these observations, we calculated the elemental content of Fe_3_O_4_ using the residual mass after the complete degradation of PLA. Consequently, the actual composite values are 18 and 33 wt.% compared to the original theoretical values of 20 and 35 wt.%. Henceforth, the samples will be referred to by these actual values.

The thermogravimetric and derivative thermogravimetric (DTG) curves for both the 18% and 33% Fe_3_O_4_ composites (Figure 6) exhibit two steps of mass loss, each with a different T_d_ compared to pure PLA. The shifts in T_d_ to lower values than those of pure PLA (18% MNP-Fe_3_O_4_/PLA: 260 °C and 33% MNP-Fe_3_O_4_/PLA: 255 °C) have also been reported in previous studies and are directly linked to the amount of magnetite added to the composite [1,14,17,55]. According to these authors, the decreased T_d_ results from the catalytic effect that magnetite exerts at elevated temperatures, facilitating the breakdown of PLA chains into lactic acid oligomers and monomers. The second step of mass loss in the composites occurs around 346 °C for the 18% MNP-Fe_3_O_4_/PLA sample and 349 °C for the 33% MNP-Fe_3_O_4_/PLA sample. This could be due to various factors, such as chemical or physical interactions between the hydroxyl groups on the magnetite surface and certain portions of the polymeric matrix [56], the aggregation of MNP-Fe_3_O_4_ leading to reduced specific surface area and catalytic activity [14], or the thermal barrier effects of the nanoparticles [20,21,24] enhancing thermal insulation and limiting the permeability of volatile degradation products [20]. Bin Xu et al. [10] similarly reported that the interaction between the polymer and MNP delays the thermal decomposition of PLA, which occurs in the temperature range of 300–400 °C, consistent with our observations.

### 3.3. Fourier-Transform Infrared Spectroscopy (FT-IR)

The pure PLA sample exhibits an absorption band near 3506 cm^−1^, associated with the stretching of the –OH bond. Adjacent bands around 3000 cm^−1^ (specifically at 2997 and 2947 cm^−1^) correspond to the symmetric and asymmetric stretches of the –CH_3_ group. A pronounced band at 1758 cm^−1^ is attributed to the stretching of the C=O bond. Additionally, the band at 1457 cm^−1^ represents the asymmetric bending of the –CH_3_ group, while the band at 1214 cm^−1^ is indicative of the asymmetric stretching of the C–O–C group. A further band at 1094 cm^−1^ corresponds to the symmetric stretching of the same C–O–C group. A band at 756 cm^−1^ is related to the bending of the C=O bond [45,46,47]. 

In the pure PLA sample, an absorption band at 3506 cm^−1^ is indicative of the characteristic terminal –OH bond found in low-molecular-weight polymers [48]. Upon the addition of 18% and 33% magnetite to the polymer matrix, a subtle shift in this band to 3502 and 3501 cm^−1^, respectively, becomes detected, indicating an interaction with MNP-Fe_3_O_4_.

**Figure 7:**
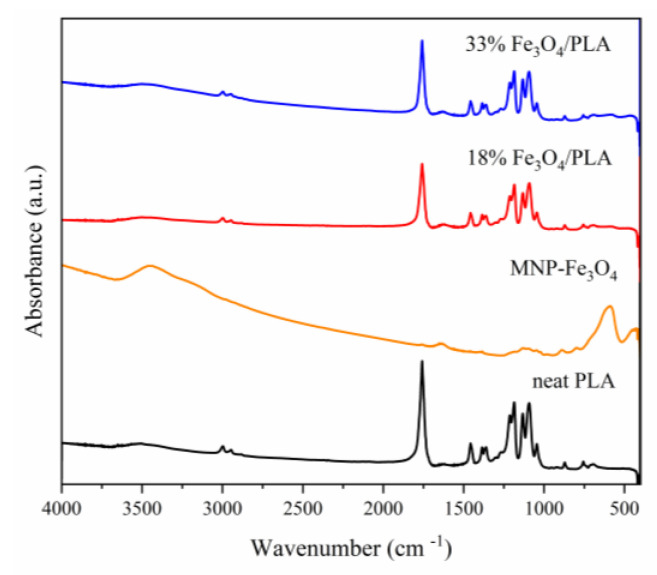
FT-IR spectra of the samples: PLA, MNP-Fe_3_O_4_, and MNP-Fe_3_O_4_/PLA composites in the proportions of 18% and 33%, respectively.

As for MNP-Fe_3_O_4_, it displays absorption bands at 442 cm^−1^ and in the range between 630 and 596 cm^−1^, which are attributed to the stretching of the Fe–O bond in iron oxide [13,54,56,57,58,59,60]. The composite materials retain both the characteristic absorption bands of PLA and those related to the Fe–O bond, which discreetly manifest between 630 and 590 cm^−1^, as shown in Figure 7.

**Figure 8:**
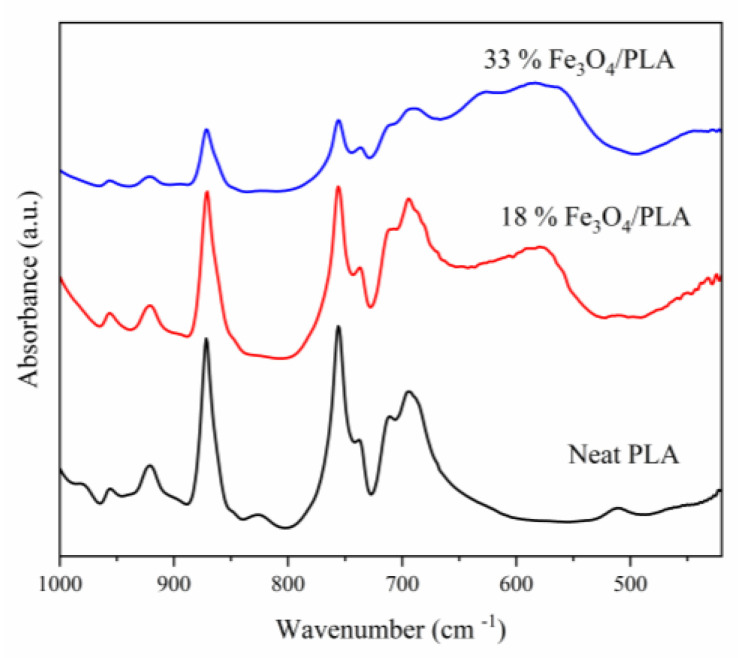
FT-IR spectra of pure PLA and MNP-Fe_3_O_4_/PLA composites made with KBr pellets (concentrated between 10% and 20% of the sample relative to the mass of KBr).

To emphasize the Fe–O bands, the FT-IR pellets were prepared with higher concentrations, specifically between 10% and 20% of the sample mass relative to KBr. The resulting spectra are depicted in Figure 8. As anticipated, the presence of Fe–O stretching bands was confirmed. These bands appeared broader and exhibited shifts compared to the pure magnetite sample, indicating interactions between the Fe–O functional group and the polymer’s functional groups. The Fe–O vibration, originally observed at 441 cm^−1^ in pure magnetite, shifts to 471 cm^−1^ and 462 cm^−1^ in the 18% MNP-Fe_3_O_4_/PLA and 33% MNP-Fe_3_O_4_/PLA composites, respectively. Furthermore, the band at 591 cm^−1^, which corresponds to Fe–O stretching in magnetite, was shifted to lower wavenumbers, i.e., specifically to 581 cm^−1^ and 585 cm^−1^ in the 18% and 33% MNP-Fe_3_O_4_/PLA composites, respectively. These shifts corroborate the interaction between MNP-Fe_3_O_4_ and PLA. 

### 3.4. Raman Spectroscopy

**Figure 9:**
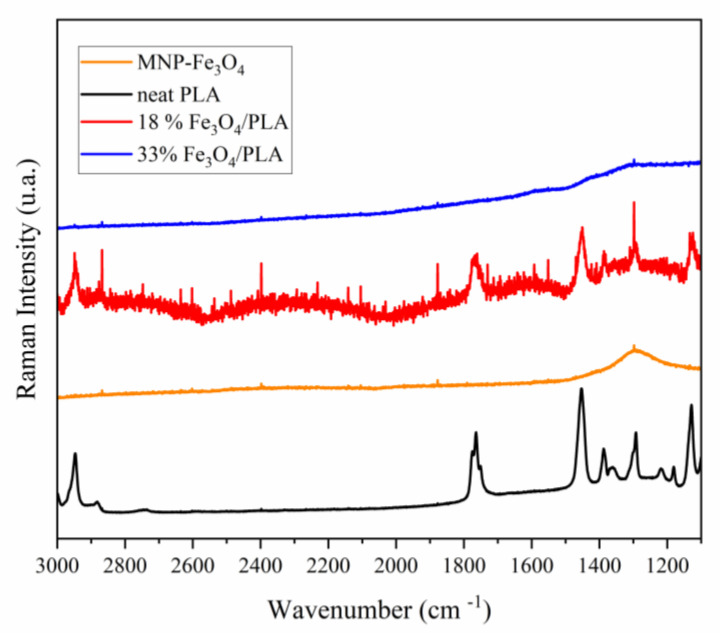
Raman spectra (3000–1100 cm^-1^) of pure PLA, MNP-Fe_3_O_4_, 18% MNP-Fe_3_O_4_/PLA, and 33% MNP-Fe_3_O_4_/PLA samples were obtained with a 795 nm laser and 90 mW of power.

The Raman spectrum of PLA (Figure 9) reveals absorption bands around 3000, 2947, and 2882 cm^−1^, attributed to the asymmetric and symmetric stretching vibrations of the C–H bond within the polymer chain. Other bands between 1775 and 1750 cm^−1^ correspond to C=O bond stretching, whereas bands at 1452 and 1128 cm^−1^ are ascribed to CH_3_ bond bending. A band at 1041 cm^−1^ is associated with C–CH_3_ bond stretching, and another at 870 cm^−1^ is ascribed to the stretching of the C–COO bond [61,62,63].

In the composite sample containing 18% MNP-magnetite, the PLA bands observed between 3000 and 870 cm^−1^ appear slightly shifted to higher wavenumbers. For instance, the bands originally observed at 1450 and 1090 cm^−1^ in PLA shift to 1453 cm^−1^ and 1094 cm^−1^, respectively, indicating the interaction between PLA functional groups and magnetite. Nonetheless, in the composite sample with a 33% MNP-Fe_3_O_4_ content, the bands corresponding to the vibrational modes inherent to PLA were not discernible. This lack of detection might be attributed to the lower polymer proportion in this composite compared to the 18% MNP-Fe_3_O_4_/PLA composite (Figure 9) or, more significantly, to the thorough coverage of micro-PLA by MNP-magnetite. Subsequent microscopy results substantiate this hypothesis, as will be explained later.

**Figure 10:**
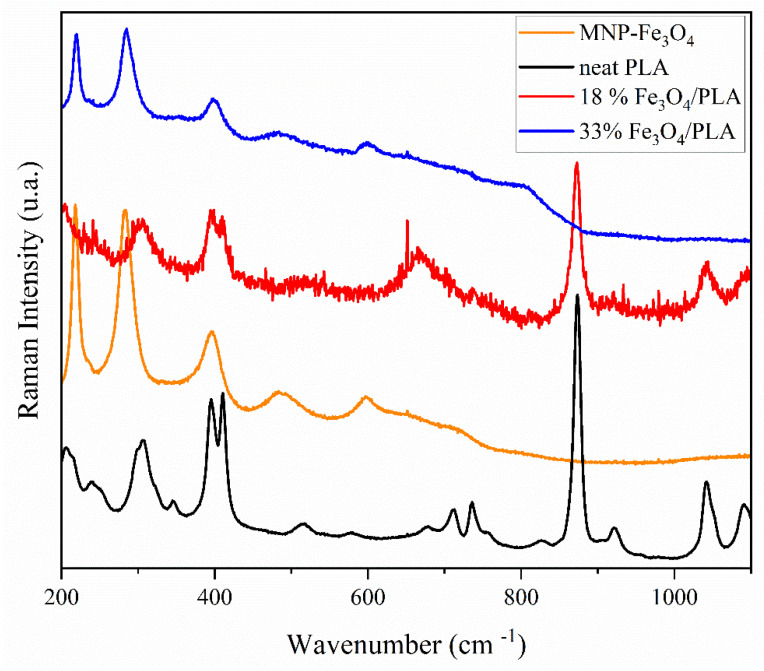
Raman spectra (1200–200 cm^-1^) of pure PLA, MNP-Fe_3_O_4_, 18% MNP-Fe_3_O_4_/PLA, and 33% MNP-Fe_3_O_4_/PLA samples were obtained with a 795 nm laser and 90 mW of power.

The spectral region between 1100 and 200 cm^−1^ encompasses the majority of vibrational modes characteristic of magnetite, maghemite, and hematite. Both maghemite and hematite can arise from the oxidation of magnetite. As demonstrated in Figure 10, the spectra of MNP-Fe_3_O_4_ and the 33% MNP-Fe_3_O_4_/PLA composite exhibit typical vibrational modes of hematite at 219, 282, 396, 485, and 597 cm^−1^ [64,65,66]. This observation suggests that the laser power employed for the analysis was excessively high, thereby causing the direct oxidation of magnetite to hematite. Conversely, the composite containing 18% MNP-Fe_3_O_4_/PLA displayed vibrations at 305, 540, and 667 cm^−1^, corresponding to the E_g_, T_2a_, and A_1g_ modes of magnetite, respectively. Additionally, a band at 514 cm^−1^ was observed, which is presumed to be characteristic of maghemite [64,65,66,67]. Given these observations, it is reasonable to infer that, in the sample with 18% MNP-Fe_3_O_4_/PLA, PLA acted as a protective layer for the magnetic nanoparticles, preventing oxidation induced by the high-power laser. The data clearly indicate that this protective effect against oxidation is dependent on the level of PLA incorporation in the composite. Mubasher et al. [68], in a recent article, indicated that magnetite functionalized by polymers can increase MNP stability and protect them in vitro and in vivo.

### 3.5. X-ray Diffraction (XRD)

**Figure 11:**
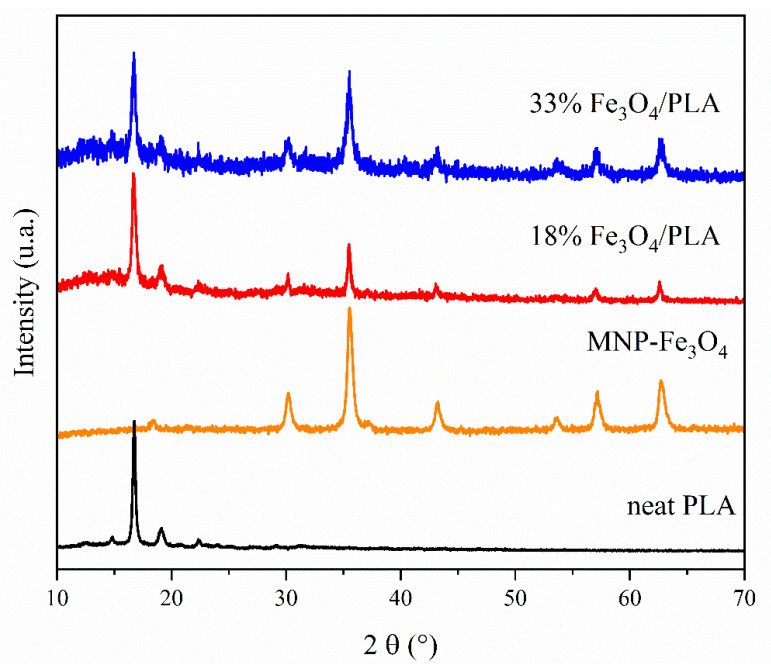
XRD patterns of the samples: pure PLA, MNP-Fe_3_O_4_, and MNP-Fe_3_O_4_/PLA composites with 18% and 33% loadings.

The X-ray diffraction (XRD) pattern exhibited by the PLA sample, as shown in Figure 11, is representative of either PLLA or PDLA crystalline structures. The semicrystalline pattern obtained for PLA was observed in the polymers synthesized with the silica-alumina catalyst, demonstrating an enantioselective character of the catalyst, as discussed in the literature [38,39]. The diffraction peaks observed at 2*θ* values of 14.7°, 16.6°, 19.1°, and 22.3° are indicative of PLLA homocrystals, whereas the peaks at 12.4° and 29.1° are characteristic of stereocomplex crystals (sc) [38,39,41,42,43]. These results are also corroborated by polarimetry measurements, which indicate an enantiomeric excess of 86% of the L-isomer in the PLA sample. Specifically, the peaks at 2*θ* = 16.6° and 19.1° correspond to the hkl planes (110/200) and (203), respectively. Similarly, the peaks at 2*θ* = 14.8° and 22.3° can be ascribed to the hkl planes (010) and (015), respectively [39].

The MNP-Fe_3_O_4_ and the composites with 18% and 33% MNP-Fe_3_O_4_/PLA content display characteristic peaks associated with the inverse spinel structure of magnetite crystals at 2*θ* values of 18.3° (111), 30.2° (220), 35.5° (311), 43.2° (400), 53.7° (422), 57.2° (511), and 62.7° (400) [1,4,13,54,69,70,71,72,73]. Notably, no peaks corresponding to the maghemite planes (211) and (210) at 2*θ* values of 26.7° and 23.7°, respectively, were detected [69,74].

These XRD findings unambiguously demonstrate the coexistence of characteristic diffraction peaks of both PLA and magnetite in the MNP-Fe_3_O_4_/PLA composites. This suggests that the crystalline structures of the individual components were preserved during the blending process.

### 3.6. Scanning Electron Microscopy (SEM)

**Figure 12:**
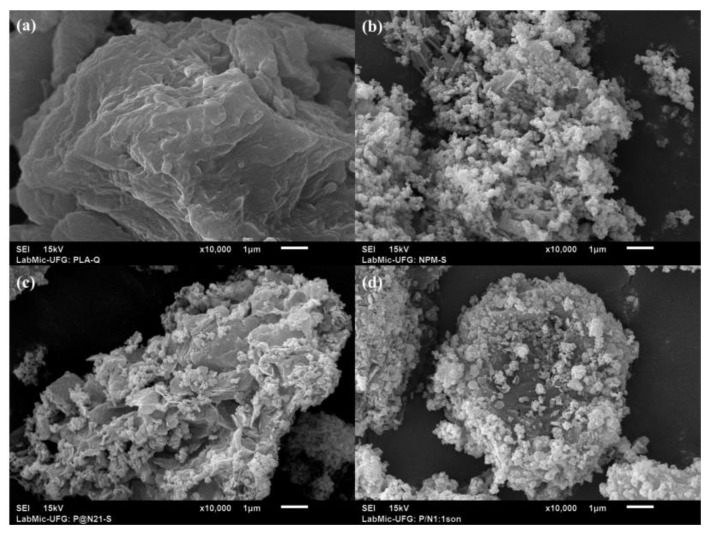
SEM micrographs of the samples: (**a**) PLA, (**b**) MNP-Fe_3_O_4_, (**c**) 18% MNP-Fe_3_O_4_/PLA, and (**d**) 33% MNP-Fe_3_O_4_/PLA.

The SEM micrographs, illustrated in Figure 12, depict a slightly rough yet homogeneous surface of a PLA block (Figure 12a). In contrast, the MNP-Fe_3_O_4_ sample features a pattern consisting of multiple, clustered spherical particles (Figure 12b). As for the composite samples, they appear to indicate the deposition and agglomeration of MNP-Fe_3_O_4_ nanoparticles onto fragments of PLA. Notably, the 18% Fe_3_O_4_/PLA composite seems to exhibit superior dispersion of nanoparticles on the polymer surface. Apparently, as seen in the SEM image of the 33% sample in Figure 12d, the magnetite nanoparticles have agglomerated and covered almost the entire surface of a micro-PLA block. This likely explains the absence of bands corresponding to the vibrational modes of PLA in the Raman spectrum of this sample. Meanwhile, the 18% MNP-Fe_3_O_4_/PLA sample shows more dispersed nanoparticles on the surface of PLA, as observed in Figure 12c (SEM). 

### 3.7. Transmission Electron Microscopy (TEM)

**Figure 13:**
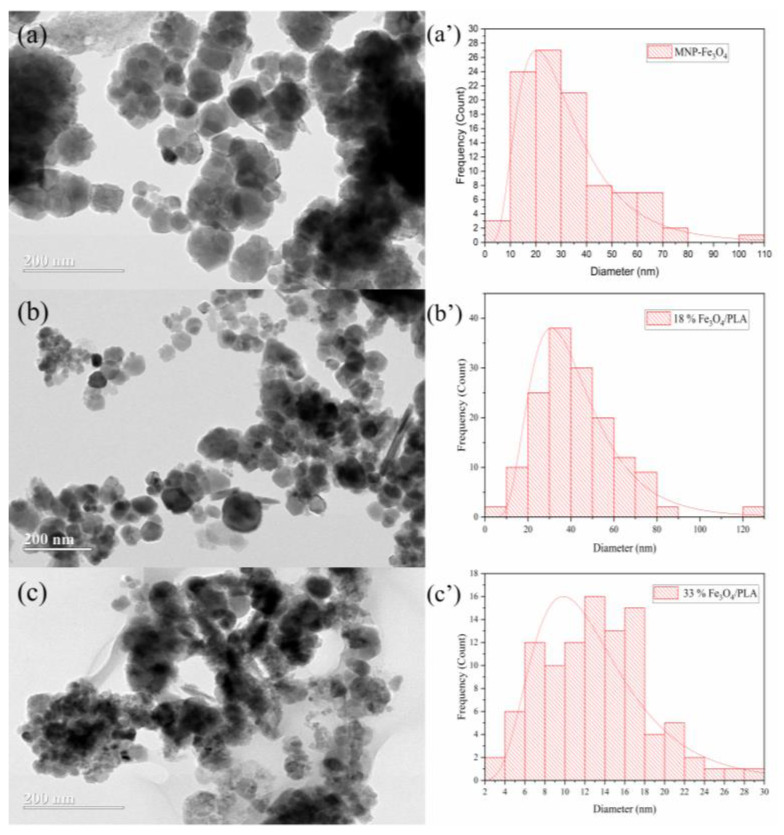
TEM micrographs of the samples: (**a**) MNP-Fe_3_O_4_; (**b**) 18% MNP-Fe_3_O_4_/PLA; and (**c**) 33% MNP-Fe_3_O_4_/PLA, and their corresponding particle size distribution histograms (**a′**–**c′**).

TEM analyses of both the MNP-Fe_3_O_4_ samples and the respective composites reveal the formation of nanoparticles with predominantly spherical morphologies, albeit with some variation in size. This size heterogeneity is particularly noticeable in the MNP-Fe_3_O_4_ sample. To ascertain the average diameter of these nanoparticles, a minimum of 100 particles were selected for quantification. The resulting size distribution is represented in the histograms presented in Figure 13. The lognormal distribution curve was applied, and the mean values of nanoparticle diameters and their corresponding standard deviations were derived from it.

Various methodologies have been explored in the literature for the synthesis of magnetite/PLA composites with differing particle size distributions. Gomez-Lopera et al. [6] and Hamoudeh et al. [7] employed the emulsion method to produce composites with average particle sizes of 180 ± 50 nm and 300 ± 10 nm to 1300 ± 180 nm, respectively. Other techniques, such as the double emulsion method employed by Wassel et al. [8], yielded particles of 85 ± 32 nm, while Nan et al. [15] utilized microwave-assisted lactide ring-opening polymerization to produce particles of approximately 23 and 27 nm in diameter. Murariu et al. [17] worked with a melting compounding technique and achieved particles with diameters around 10 and 20 nm. Tudorachi et al. [58] and Yao et al. [21] also reported a range of particle sizes using different methods of 420 to 864 nm and 150 to 700 nm in diameter, respectively.

Contrastingly, in the current study, MNP-Fe_3_O_4_ nanoparticles and their composites were successfully synthesized with diameters ranging from 12 to 38.2 nm, as summarized in Table 3. Significantly, these results were achieved without employing conventional synthesis methods like emulsion or fusion techniques. Additionally, neither magnetite nor PLA underwent any surface treatment. This underscores the utility of sonochemical synthesis as a promising route for obtaining nanoparticle materials. Specifically, this method yielded particles with well-defined spherical morphologies and fair dispersion properties.

The observation that the 18% Fe_3_O_4_/PLA composite exhibited a larger average particle diameter compared to pure magnetite is consistent with previous literature. Tudorachi et al. [58] have similarly suggested that the increase in particle size in such composites is attributable to the polymeric coating encasing the magnetite particles. The coating is likely to have contributed to the observed increase in particle diameter due to the overlap of magnetite particles by the PLA.

Furthermore, the presence of this PLA coating appears to have functional benefits beyond merely altering particle size. The Raman analysis indicates that the 18% MNP-Fe_3_O_4_/PLA composite is more resistant to oxidation compared to pure magnetite. This is an important attribute, especially in applications where oxidative stability is a critical parameter. The apparent coating of PLA, discernible as white contrast layers around the nanoparticles, supports this hypothesis. This coating can act as a barrier that minimizes the susceptibility of magnetite to environmental factors, i.e., enhancing its oxidative stability.

**Figure 14:**
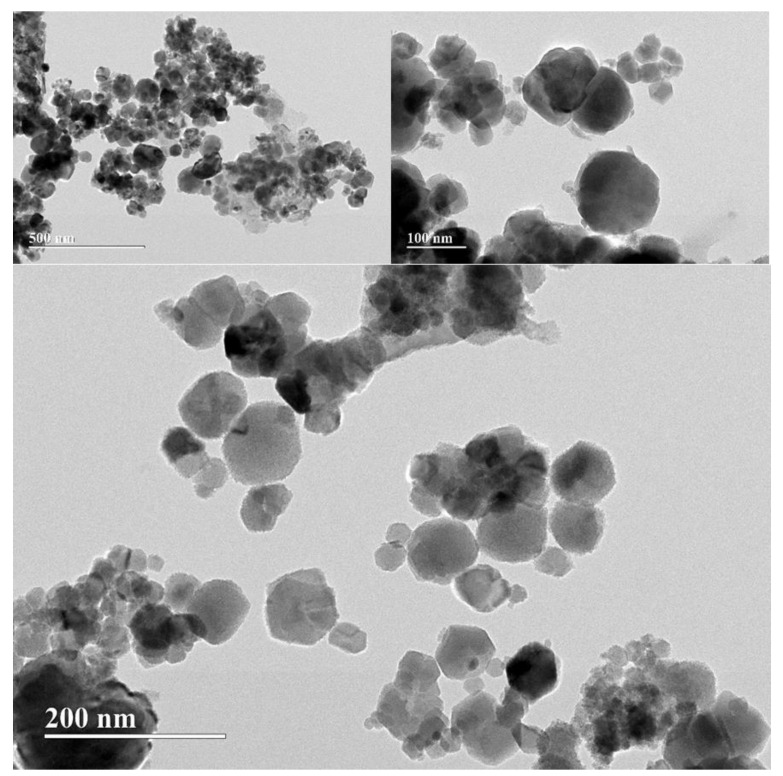
TEM micrographs of the 18% MNP-Fe_3_O_4_/PLA sample at different levels of magnification: 500, 200, and 100 nm.

The visual evidence provided in Figure 14, showing the 18% MNP-Fe_3_O_4_/PLA composite at different magnification levels, further corroborates the role of PLA as a possible protective layer. The well-defined particles, in conjunction with the apparent PLA coating, substantiate the idea that the polymer plays a pivotal role in shielding the magnetite from oxidation, as revealed in the Raman analysis.

In general, the multi-faceted utility of the PLA coating, ranging from influencing particle size to enhancing oxidative stability, underscores the synergistic benefits of this composite material. The findings offer valuable insights into the interplay of structure and function in MNP-Fe_3_O_4_/PLA nanocomposites, providing possibilities for future research and potential applications.

The findings related to the sample of 33% MNP-Fe_3_O_4_/PLA are in line with previous studies suggesting a dependence of particle size and oxidation resistance on the ratio of magnetite to PLA. The observation of smaller and more homogeneous particle sizes in the 33% sample is notable, but the decreased resistance to laser-assisted oxidation during Raman analysis is a concern. This discrepancy can be elucidated by referring to the work by Zhao et al. [9], who indicated that high magnetite loading may result in insufficient PLA to form a protective coating around the nanoparticles.

**Figure 15:**
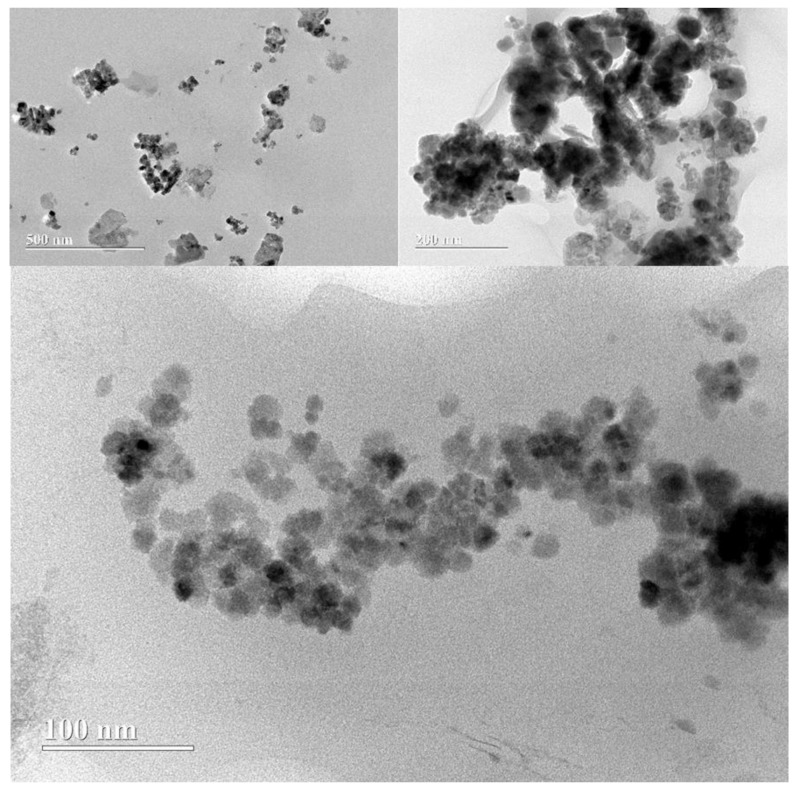
TEM micrographs of the 33% MNP-Fe_3_O_4_/PLA sample at different levels of magnification: 500, 200, and 100 nm.

The structure described in Figure 15, where PLA appears to serve as a matrix hosting magnetite nanoparticles in a “sea-island” type arrangement, further underscores the hypothesis of weak adhesion between the PLA and magnetite phases. As per Yu et al. [1], this structure becomes increasingly evident with higher magnetite loads. This morphological attribute could contribute to the observed reduction in oxidative resistance in the Raman analysis for the 33% MNP-Fe_3_O_4_/PLA nanocomposite. This is consistent with the coverage of MNP-Fe_3_O_4_ on the PLA block, as observed in Figure 12d (SEM), which likely exposes the magnetic nanoparticles more and makes them susceptible to oxidation.

Therefore, while a high magnetite load may offer advantages such as smaller particle sizes, it also appears to come with trade-offs, particularly in terms of oxidative stability. This nuanced behavior offers significant implications for the design and application of magnetite-PLA nanocomposites, particularly in scenarios where both particle size uniformity and oxidative stability are paramount.

Overall, these results serve as an instructive lesson in the complex interplay between composition, structure, and functional properties in nanocomposite materials. Future research could focus on optimizing this balance to create composites that effectively match the benefits of both components while minimizing their respective limitations.

### 3.8. Elemental Analysis by Energy-Dispersive X-ray Spectroscopy (EDX)

**Figure 16:**
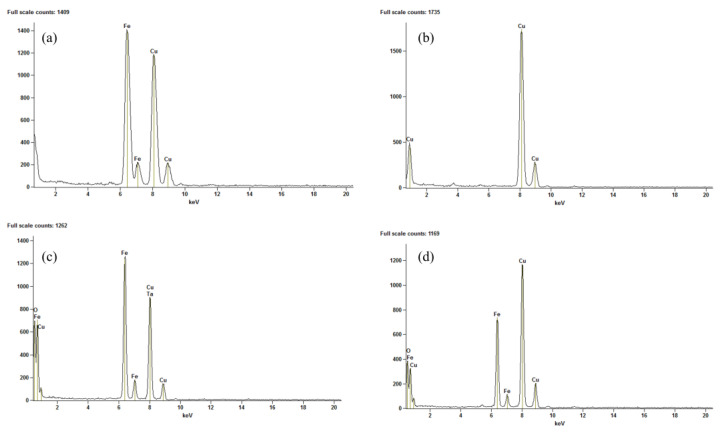
EDX elemental analysis of (**a**) MNP-Fe_3_O_4_; (**b**) PLA; (**c**) 18% MNP-Fe_3_O_4_/PLA; and (**d**) 33% MNP-Fe_3_O_4_/PLA.

The EDX elemental spectra, as depicted in Figure 16, confirm the presence of iron and oxygen in the MNP-Fe_3_O_4_, 18% MNP-Fe_3_O_4_/PLA, and 33% MNP-Fe_3_O_4_/PLA samples. Additionally, the PLA sample reveals no peaks for elements other than copper, which was utilized during the sample preparation. It is worth mentioning that carbon was not detected by the EDX system employed in our study. This observation substantiates that the materials remained uncontaminated by impurities (e.g., residues of catalyst) throughout the polymer synthesis process.

Thus, we can quickly point out some of the main achievements. The composites synthesized via sonochemistry exhibited a thermal degradation profile with two stages of mass loss, one at a higher and the other at a lower T_d_ compared to pure PLA. The mass loss stage occurring around 350 °C is likely related to the part of PLA that reacts chemically with magnetite, such as through hydrogen bonds. FT-IR results support this hypothesis, as it is possible to identify the shifting of the terminal –OH bands of the PLA chain and bands related to the Fe–O bond, suggesting a connection between these groups. Morphological analysis (TEM) of the 33% MNP-Fe_3_O_4_/PLA composite, which displayed a sea-island structure, suggests weak adhesion between magnetite and the polymer phase, in agreement with the FT-IR results. Conversely, the results presented by the 18% MNP-Fe_3_O_4_/PLA sample suggest that the PLA at least partially coated the nanoparticles rather than acting as a matrix where they would aggregate. This assumption, in turn, is supported by the Raman results, where the 18% MNP-Fe_3_O_4_/PLA composite showed resistance to oxidation, exhibiting the expected bands for magnetite, while the uncoated MNP-Fe_3_O_4_ and the 33% MNP-Fe_3_O_4_/PLA composite were completely oxidized to hematite, demonstrating that in one material, PLA acted as a protective layer, while in the other, it did not. Also, XRD results showed that all synthesized materials maintained their crystalline structures after blending, with the magnetite peaks less pronounced in the sample with an 18 wt.% loading, which may be related to the lower MNP-Fe_3_O_4_ content or the effect of the PLA coating.

## 4. Conclusions

Sonochemical synthesis has proven to be an alternative for producing MNP-Fe_3_O_4_/PLA nanocomposites. The synthesized nanocomposites exhibited notable thermal properties, characterized by two distinct stages of mass loss: one at a lower degradation temperature (T_d_) compared to pure PLA, and another at a higher T_d_. This suggests that the interaction between PLA and MNP-Fe_3_O_4_ may occur through hydrogen bonds, leading to enhanced thermal stability of a segment of the polymer. This hypothesis was corroborated by Fourier Transform Infrared (FT-IR) analysis, which showed shifts in bands related to the terminal –OH groups of the polymer and Fe–O bonds, thereby confirming the interaction between these groups. Raman spectroscopy results demonstrated that PLA serves as a protective layer against the oxidation of MNP-Fe_3_O_4_ in the 18% MNP-Fe_3_O_4_/PLA nanocomposite when exposed to a high-power laser (90 mW). Microscopic analyses (SEM and TEM) indicated that the proposed synthetic method, in the case of composites, formed dispersed nanoparticles within the PLA matrix, even in the absence of surfactants or stabilizers. Energy-Dispersive X-ray (EDX) elemental analysis revealed that both MNP-Fe_3_O_4_ and the composites primarily contain iron and oxygen, while the PLA sample exhibited no detectable impurities.

## Figures and Tables

**Table 1:** Some examples of reports involving Fe_3_O_4_/PLA composites.

Ref.	Modification on Magnetite or PLA	Composite Synthesis Technique	Solvents	Fe_3_O_4_ (wt.%)
[10]	Polyethylene glycol-PLA copolymer (MEPLEG)	Double emulsion with simultaneous coprecipitation of Fe(II) and Fe(III)	CH_2_Cl_2_ organic phase; PVA water phase	0–43
[11]	PLGA copolymer; Fe_3_O_4_ functionalized with oleic acid	Double emulsion with solvent evaporation	CH_2_Cl_2_ organic phase; PVA water phase	1
[12]	PLAU copolymer (PLA-based polyurethane); Fe_3_O_4_ functionalized with oleic acid	Emulsion	CH_2_Cl_2_	0–9
[13]	PLA-b-PEG copolymer; Aldehyde modified Fe_3_O_4_	UGI type condensation; composite microspheres obtained by simple emulsion	CH_2_Cl_2_ organic phase; PVA water phase	Not specified
[14]	Fe_3_O_4_ functionalized with oleic acid	mixture of ethanol solution with PLA and magnetite dispersed by ultrasound	Ethanol	0–16
[17]	Fe_3_O_4_ treated with 3% polymethylhydrogen-siloxane	Melting compound	No solvent	4–16
[18]	Non-functionalized	Extrusion	No solvent	20
[19]	Non-functionalized	Doctor blade technique	CH_3_Cl	1–10
[22]	Fe_3_O_4_ functionalized with ricinoleic acid	Solvent-casting method for the preparation of Fe_3_O_4_ capped PLA	THF and CH_2_Cl_2_	~25
[23]	Non-functionalized	Casting	CH_2_Cl_2_	1
[24]	Fe_3_O_4_ functionalized with SiO_2_ and B-cyclodextrin	Solvent-casting	CH_3_Cl	0–8
[25]	Fe_3_O_4_ conjugated with eucalyptus essential oil	Matrix-assisted pulsed laser evaporation (MAPLE) technique	Dimethyl sulfoxide (DMSO)	3

**Table 2:** Results of the molar masses obtained by GPC of the polymers synthesized from D,L-lactic acid, where M_n_ is the average molar mass, M_w_ is the average molar mass, and M_w_/M_n_ is the molar mass distribution index.

Sample	M_n_ (Da)	M_w_ (Da)	M_w_/M_n_
Pre-polymer	2278	2705	1.2
PLA (without catalyst)	5830	7049	1.2
PLA 1x	6930	8702	1.2
PLA 2x	6835	9111	1.3
PLA 3x	6937	9634	1.4

**Table 3:** The average particle diameter (nm) is calculated from TEM measurements.

Sample	Average Particle Diameter (nm)
MNP-Fe_3_O_4_	27.9 ± 1.7
18% MNP-Fe_3_O_4_/PLA	38.2 ± 1.6
33% MNP-Fe_3_O_4_/PLA	12.0 ± 1.5

## Data Availability

The data presented in this study are available on request from the corresponding authors.
